# Primary vs. pre-emptive anti-seizure medication prophylaxis in anti-CD19 CAR T-cell therapy

**DOI:** 10.1007/s10072-024-07481-0

**Published:** 2024-03-21

**Authors:** Umberto Pensato, Federica Pondrelli, Chiara de Philippis, Gian Maria Asioli, Alessandra Crespi, Alessandro Buizza, Daniele Mannina, Beatrice Casadei, Enrico Maffini, Laura Straffi, Simona Marcheselli, Pier Luigi Zinzani, Francesca Bonifazi, Maria Guarino, Stefania Bramanti

**Affiliations:** 1 Department of Biomedical Sciences, Humanitas University, Via Rita Levi Montalcini 4, 20072 Pieve Emanuele, Milan, Italy; 2 IRCCS Humanitas Research Hospital, Via Manzoni 56, 20089 Rozzano, Milan, Italy; 3 Department of Biomedical and Neuromotor Sciences (DIBINEM), University of Bologna, Bologna, Italy; 4 IRCCS Istituto Delle Scienze Neurologiche Di Bologna, Bologna, Italy; 5 BMT and Cell Therapy Unit, Humanitas Cancer Center, IRCCS Humanitas Research Hospital, Rozzano, Milan Italy; 6 IRCCS Azienda Ospedaliero-Universitaria Di Bologna, Istituto Di Ematologia “Seràgnoli”, Bologna, Italy; 7 Dipartimento Di Scienze Mediche E Chirurgiche, Università Di Bologna, Bologna, Italy

**Keywords:** Status epilepticus, Neurotoxicity, Electroencephalography (EEG), Immune effector-associated neurotoxicity syndrome (ICANS), Anti-epileptic drugs, Cancer immunotherapy, Neurological complications, Non-Hodgkin lymphoma, Cytokine storm-associated encephalopathy (CySE)

## Abstract

**Introduction:**

Seizures may occur in up to 30% of non-Hodgkin lymphoma patients who received anti-CD19 CAR T-cell therapy, yet the optimal anti-seizure medication (ASM) prevention strategy has not been thoroughly investigated.

**Methods:**

Consecutive patients affected by refractory non-Hodgkin lymphoma who received anti-CD19 CAR T-cells were included. Patients were selected and assessed using similar internal protocols. ASM was started either as a primary prophylaxis (PP-group) before CAR T-cells infusion or as a pre-emptive therapy (PET-group) only upon the onset of neurotoxicity development.

**Results:**

One hundred fifty-six patients were included (PP-group = 88, PET-group = 66). Overall, neurotoxicity and severe neurotoxicity occurred in 45 (29%) and 20 (13%) patients, respectively, equally distributed between the two groups. Five patients experienced epileptic events (PET-group = 3 [4%]; PP-group = 2 [2%]). For all the PET-group patients, seizure/status epilepticus occurred in the absence of overt CAR-T-related neurotoxicity, whereas patients in the PP-group experienced brief seizures only in the context of critical neurotoxicity with progressive severe encephalopathy. ASMs were well-tolerated by all patients, even without titration. No patients developed epilepsy or required long-term ASMs.

**Conclusion:**

Our data suggest that both primary and pre-emptive anti-seizure prophylaxis are safe and effective in anti-CD19 CAR T-cell recipients. Clinical rationale suggests a possible more favourable profile of primary prophylaxis, yet no definitive conclusion of superiority between the two ASM strategies can be drawn from our study.

**Supplementary Information:**

The online version contains supplementary material available at 10.1007/s10072-024-07481-0.

## Introduction

Chimeric antigen receptor (CAR) T-cell therapy represents a novel and highly effective treatment for refractory haematological cancers [1, 2]. Yet, it is often complicated by systemic and neurological toxicities, namely cytokine release syndrome (CRS) and immune effector-associated neurotoxicity syndrome (ICANS) [3, 4]. Neurotoxicity usually develops concomitantly or a few days after CRS and arguably results from cytokine-mediated neuroinflammation [3, 5–7].

ICANS may present in up to 77% of the patients and has a monophasic, fluctuating, or progressive, acute course [8]. Neurological manifestations are heterogeneous and include dysgraphia, non-fluent aphasia, tremors, headache, frontal lobe dysfunction, altered mental status, seizures, and status epilepticus [8–16]. Notably, the incidence of seizures among patients undergoing CAR T-cell therapy, despite being highly heterogeneous, is reported in up to 30% [8, 9, 17]. Additionally, electroencephalographic abnormalities are almost ubiquitously observed during neurotoxicity [18, 19]. Therefore, anti-seizure medications (ASMs) have been employed in preventive regimens in clinical trials and real-world studies on CAR T-cell recipients, yet it is not clear which is the best strategy to adopt in terms of efficacy and risk of adverse effects [9]. This study aims to explore the efficacy and safety of ASMs implementation in two real-world cohorts adopting different (primary and pre-emptive) seizures prophylaxis strategies in non-Hodgkin lymphoma patients treated with anti-CD19 CAR T-cells.

## Methods

### Standard protocol approval, registrations, and patient consent

The study was approved by the local institutional review boards (protocol number: CE: 319/2021/Sper/AOUBo and ONC/OSS-02/2022). Written informed consent was obtained from all enrolled patients for study participation and data publication. All procedures were conducted according to the latest version of the Declaration of Helsinki.

### Study design

This was a multicentric, real-life cohort study. Patients affected by large B-cell non-Hodgkin lymphoma (diffuse large B-cell lymphoma [DLBCL], primary mediastinal B-cell lymphoma [PMBCL], mantle cell lymphoma [MCL], and follicular lymphoma [FL]) who received anti-CD19 CAR T-cell therapy were prospectively enrolled from August 2019 to March 2023. Two patients presenting with fulminant cerebral oedema were excluded from the study due to the exceptionally severe nature of this condition and its high likelihood of rapidly progressing to a fatal outcome despite aggressive treatment interventions. Patients were recruited from two haematological centres in Italy: IRCCS Policlinico Sant’Orsola-Malpighi in Bologna and IRCCS Humanitas Research Hospital in Milan. Patients were selected and assessed with similar internal protocols, except for ASMs initiation. Indeed, at the former centre, all patients received primary prophylaxis (PP-group)—ASM introduced before CAR T-cell infusion. In contrast, at the latter centre, ASM was administered as either primary prophylaxis (PP-group) or as pre-emptive therapy (PET-group) at the discretion of the treating haematologist. In the pre-emptive regimen, ASMs were administered only upon evidence of neurotoxicity development (ICANS ≥ 1). The standard ASM employed was Levetiracetam 750 mg twice daily in both groups with dosage adjustment based on drug plasmatic levels. Levetiracetam was chosen for seizure prophylaxis due to its favourable drug-drug interaction profile and reduced risk of cardiotoxicity in comparison to other anti-seizure medications [20]. Additionally, it can be safely administered to patients with hepatic dysfunction, although dosage adjustments might be necessary for individuals with renal dysfunction [20]. Moreover, it is noteworthy that levetiracetam treatment does not impact cytokine levels, further underscoring its safety in CAR T-cell recipients [21].

The ASM was slowly tapered and discontinued after one month from CAR T-cell infusion for patients who did not experience any seizures or at the discretion of the treating neurologist for the patients who had seizures.

The same inclusion criteria, defined by the Italian Medicine Agency (AIFA) reimbursement criteria, were applied in both centres. These included the following: (i) > 18 years old, (ii) diagnosis of non-Hodgkin lymphoma refractory or relapsed after at least two specific anti-tumour therapies, (iii) absence of a poor performance status (defined by an Eastern Cooperative Oncology Group Performance Status [ECOG PS] > 1), (iv) absence of central nervous system involvement, and (v) absence of severe organ dysfunction. A brain MRI with contrast was performed on every patient to exclude central nervous system involvement. Additionally, on a case-by-case basis, a neurological examination and diagnostic lumbar puncture were also performed in the presence of red flags.

The haematological protocol adopted by the two centres was equivalent. Following leukapheresis, the patients received variable bridging therapies at the discretion of the treating haematologist to restrain the cancer burden while waiting for CAR T-cell manufactory and infusion. The bridging therapies adopted included chemotherapy, radiotherapy, corticosteroids, or immunotherapy. As a lymphodepleting chemotherapy regimen, all patients received cyclophosphamide (250–500 mg/m^2^) and fludarabine (25–30 mg/m^2^) for three days. All patients received an anti-CD19 CAR t-cell product: axicabtagene ciloleucel (axi-cell), tisagenlecleucel (tisa-cel), or brexucabtagene autoleucel (brexu-cel).

### Toxicity assessment and management

CRS and ICANS were diagnosed and graded according to the American Society for Transplantation and Cellular Therapy (ASTCT) Consensus Grading [3]. The neurological assessment protocol was slightly different in the two centres. In the PP-group, EEG and neurologist consultations were performed every other day since CAR T-cell infusion, whereas in the PET-group, neurologist consultations and EEG were performed only after neurotoxicity onset. Detailed information on the neurological assessment and management at the IRCCS Malpighi-Sant’Orsola Hospital has been previously published [11]. In both groups, every 6 h since CAR T-cell infusion, examinations to detect neurotoxicity signs were performed by health personnel (haematologists or nurses) who received specific training in the assessment of ICANS and always included the ICE (immune effector cell-associate encephalopathy) score. Upon evidence of neurotoxicity development, neurological diagnostic tests were performed at the discretion of the treating neurologist, including brain MRI, standard or continuous EEG, diagnostic lumbar puncture, and brain FDG-PET. Conversely, neurotoxicity management was homogenous between centres. Intravenous dexamethasone 10 mg three or four times a day was started in the case of grade 2 ICANS. Whenever a severe (grade ≥ 3) neurotoxicity was diagnosed, intravenous high-dose methylprednisolone (1000 mg daily for 3–5 days) was promptly started, and the patient was transferred to an intensive care unit. Finally, in the case of steroid-refractory neurotoxicity, defined as the clinical progression or absence of amelioration following 48 h of high-dose steroids, monoclonal anti-cytokines antibodies were administered (anakinra or siltuximab). In summary, the neurotoxicity surveillance was more scrupulous before ICANS onset in the PP-group, whereas neurotoxicity follow-up and management overlaid.

### Outcomes

The primary outcome of the study was to evaluate the safety and efficacy of the two ASM protocols, defined by drug tolerance, and the seizure and status epilepticus incidence. The secondary outcomes were to characterize the clinical and EEG features of the epileptic manifestations and describe their management and long-term follow-up.

### Statistics

The statistical analysis was performed with IBM SPSS Statistics Version 29. Continuous variables were reported as mean and standard deviation and assessed for distribution with the Kolmogorov–Smirnov normality test. Normally distributed variables were reported with the paired sample *t*-test, whereas not normally distributed variables with the Wilcoxon signed-rank test. Categorical variables were expressed as counts and percentages and compared with the Pearson Chi-square test. All calculated *p* values were two-tailed. Statistical significance was set at *p* < 0.05.

## Results

### Patient disposition, baseline characteristics, and neurotoxicity features

Comprehensively, 156 patients were enrolled in the study: 88 patients in the PP-group (75 from IRCCS Malpighi Sant’Orsola Hospital and 13 from IRCCS Humanitas Research Hospital) and 68 patients in the PET-group (all from IRCCS Humanitas Research Hospital). The epidemiological and clinical characteristics of the two groups are summarized in Table 1. The average age was 60, and 54 (35%) patients were females. Most patients were affected by DLBCL (72%) and had an advanced disease stage at the time of CAR T-cell infusion (74%). Different CAR T-cell products have been administered: tisa-cel (51%), axi-cel (37%), and brexu-cel (12%). Most of the patients (83%) experienced CRS, yet severe systemic toxicity was rare (6%). Overall, ICANS and severe ICANS occurred in 45 (29%) and 20 (13%) patients, respectively, equally distributed between the two groups. Yet, the time of neurotoxicity onset was slightly delayed in the PET-group (8.2 days vs 5.2 days; *p* = 0.081). The PP-group presented a higher mean number of failed therapies for haematological disease (2.83 vs. 2.26; *p* = 0.003) and a longer cancer history (47 vs. 30 months; *p* = 0.031). No other statistically significant difference in baseline epidemiological or clinical features was observed between the two groups.

**Table 1:** Comparison of baseline and neurotoxicity features in the two cohorts

	Total patients (*n* = 156)	PP-group (*n* = 88)	PET-group (*n* = 68)	*P* value
Age (years) (mean ± SD)	55.97 (13.58)	55.95 (13.45)	55.81 (13.01)	0.855
Female sex	54 (35%)	27 (31%)	27 (40%)	0.801
Histology subtypes				
DLBCL	113 (72%)	66 (75%)	47 (69%)	0.666
PMBCL	23 (15%)	11 (13%)	12 (15%)	0.340
MCL	19 (12%)	10 (11%)	9 (13%)	0.364
FL	1 (1%)	1 (1%)	0 (0%)	-
Bulky (> 7 cm)	62 (40%)	38 (43%)	24 (35%)	0.564
Bridging therapy with pembrolizumab	17 (11%)	7 (8%)	10 (15%)	0.351
Number of previous treatments (mean ± SD)	2.58 (1.20)	2.83 (1.29)	2.26 (0.75)	**0.003**
Prior ASCT	41 (26%)	28 (32%)	13 (19%)	0.790
History of lymphoma disease (months) (mean ± SD)	39.42 (53.86)	47.03 (58.07)	29.57 (46.44)	**0.031**
Disease stages III–IV	116 (74%)	66 (75%)	50 (73%)	0.214
CAR T-cells product				
Axicabtagene ciloleucel	58 (37%)	40 (45%)	18 (26%)	0.707
Tisagenlecleucel	79 (51%)	41 (46%)	38 (56%)	0.400
Brexucabtagene autoleucel	19 (12%)	10 (11%)	9 (13%)	0.240
CRS				
CRS incidence	129 (83%)	73 (83%)	56 (82%)	0.568
Severe CRS incidence	9 (6%)	4 (5%)	5 (7%)	0.618
Neurotoxicity				
ICANS incidence	45 (29%)	25 (28%)	20 (29%)	0.417
Severe ICANS incidence	20 (13%)	10 (11%)	10 (15%)	0.887
Time at onset (days) (mean ± SD)	6.53 (5.08)	5.20 (3.63)	8.20 (6.14)	0.081
Seizure incidence	5 (3%)	2 (2%)	3 (4%)	0.829
Status epilepticus incidence	3 (2%)	1 (1%)	2 (3%)	-

*ICANS* immune effector cell-associated neurotoxicity syndrome, *ASCT* autologous stem cell transplantation, *EEG* electroencephalography, *DLBCL* diffuse large B-cell lymphoma, *PMBCL* primary mediastinal B-cell lymphoma, *MCL* mantle cell lymphoma

### Seizure and status epilepticus incidence, clinical characteristics, timing, and management

Five patients (3%) experienced seizures, three from the PET-group (4%) and two from the PP-group (2%). Three patients progressed to a status epilepticus. Detailed clinical characteristics of the five patients are reported in Table 2 and Supplementary information. No statistical comparison to assess for prognostic factors associated with seizure development was possible due to the small sample size. Three patients were young women (31–32 years old) affected by PMBCL, received pembrolizumab as a bridge therapy, and received Axi-cel as the CAR T-cell product. Notably, the three patients in the PET-group exhibited seizure/status epilepticus as their primary and sole neurotoxicity manifestations, even though all patients had already experienced CRS. Conversely, the two patients in the PP-group encountered epileptic events following the onset of extremely severe neurotoxicity (ICANS grade 4): one patient experienced a single seizure, while the other encountered three clustered brief seizures.

**Table 2:** Epidemiological and clinical features of patients who experienced seizures

	Pt 1 (PET-group)	Pt 2 (PET-group)	Pt 3 (PET-group)	Pt 4 (PP-group)	Pt 5 (PP-group)
Age (y)	31	52	31	33	63
Sex	F	M	F	F	M
Haematological disease	PMBCL	DCBCL	PMBCL	PMBCL	DLBCL
CAR T-cell product	Axi-cel	Tisa-cel	Axi-cel	Axi-cel	Axi-cel
CRS max grade	1	1	1	2	1
First neurological manifestation	Status epilepticus	Seizure	Status epilepticus	Aphasia	Aphasia
Time of neurological onset (days from CAR T-cell infusion)	5	14	7	4	4
ICANS max grade (excluding seizure)	0	0	0	4	4
Seizure description	Super-refractory non-convulsive status epilepticus	Tonic–clonic seizure	Refractory non-convulsive status epilepticus	Tonic–clonic seizure	Tonic–clonic status epilepticus
Timing of seizure onset from neurotoxicity onset	0	0	0	20 h	4 h
Seizure alone	Yes	Yes	Yes	No	No
ICANS treatment	Steroid, anakinra	Steroid	Steroid	Steroid, anakinra, siltuximab	Steroid, siltuximab
Seizure treatment	Three ASMs + anaesthetics	Start ASM	Start ASM	Increase ASM	Increase ASM
Neurotoxicity resolution timing (days)	11	1	2	5	5
Hospitalization duration (days from CAR T-cell infusion)	40	21	20	14	28
ICU admission indication	Refractory status epilepticus	No	Status epilepticus	Decrease level of consciousness	Severe ICANS (according to internal protocol)
ICU duration (days)	11	0	3	6	3
Haematological outcome	Complete response	Complete response	Complete response	Complete response	Partial response

*CRS* cytokine release syndrome, *ICANS* immune effector cell-associated neurotoxicity syndrome, *ICU* intensive care unit, *PMBCL* primary mediastinal B-cell lymphoma, *DLBCL* Diffuse large B-cell lymphoma, *ASM* anti-seizure medication

### Safety and long-term follow-up

No patient experienced ASM intolerance in the PP-group where treatment was slowly titrated. In the PET-group, no apparent drug intolerance was observed, yet the neurotoxicity manifestations presented concomitantly, hampering an accurate evaluation of possible iatrogenic symptoms. In all the PP-group patients, levetiracetam plasma concentrations were in the normal range at neurotoxicity onset. No patients experienced any further seizures at follow-up or required the maintenance of ASM.

## Discussion

Seizures and status epilepticus were relatively rare in our study compared to historical cohorts, with a prevalence of 4% and 2%, respectively, suggesting the effectiveness of both prophylaxis strategies. Two patients had isolated tonic–clonic seizures, whereas three patients developed a status epilepticus. Notably, these three patients were young women affected by PMBCL and received pembrolizumab as the bridge therapy. Axi-cel products and immune checkpoint inhibitors before infusion are well-known risk factors for developing CAR T-cell toxicities [22]. Conversely, the potential role of PMBCL and the female sex as potential risk factors for seizure and neurotoxicity at large remains unexplored. Future more extensive studies to investigate which of the abovementioned features is the main driver risk factor are warranted for definitive conclusions. Yet, in the meanwhile, considering that these epidemiological features and therapeutic options are likely to present combined—PBMCLs usually present in young females treated with Axi-cel [23]—they should be considered at a high risk of epileptic events.

Even though the incidence of seizure in the primary prophylactic group was only minimally inferior compared to the pre-emptive group, some critical clinical differences were observed. Notably, the patients from the primary prophylaxis group experienced seizure only after developing severe neurotoxicity. Conversely, all three patients from the PET group displayed seizure and status epilepticus in the absence of signs of broader neurotoxicity. Taken together, these data suggest that the use of prophylactic ASMs might have the potential to effectively mitigate seizures when they are the exclusive neurotoxicity presentation, while also potentially attenuating the severity of epileptic manifestations in fully developed ICANS. Moreover, a gradual titration of ASM could serve as a protective measure against the risk of neurological side effects associated with rapid intravenous bolus titration, which is essential in the PET group to promptly achieve the therapeutic range.

Additionally, while an epileptic event might not have an immediate bearing on the prognosis, even an isolated seizure can increase the duration of hospitalization in the intensive care unit and/or the haematology ward. Consequently, this prolonged hospital stay heightens the susceptibility of immunocompromised patients to infections and may amplify the economic burden associated with their care [24].

Interestingly, patients in the PET group who experienced seizures showed a delayed onset of neurotoxicity compared to those in the PP group. Considering that these patients did not present any other neurotoxicity manifestations, this observation might reflect a heightened brain susceptibility to seizures in the days or weeks following CAR T-cell infusion, even in the absence of overt neurotoxicity in patients not receiving anti-seizure medications. Conversely, seizure patients in the PP group were observed in the context of severe neurotoxicity, that usually occur in the first days following CAR T-cells infusion. However, the limited number of cases prevent to draw any definitive conclusion.

Notably, no patients developed further seizures during the follow-up, supporting the transient nature of the seizure risk factors without persistent sequelae. This is in line with mounting recent evidence that supports the reversibility of neurotoxicity and long-term neurologic safety following CAR T-cell therapies [11, 25, 26]. Additionally, no patient experienced any drug intolerance with both ASM regimens. Yet, we cannot exclude that patients in the PET group may have developed some drug-related side effects that were not detected due to the progression of neurotoxicity symptoms [27]. Indeed, the ASM was started only after the onset of neurological manifestations, mainly encephalopathy, and with no drug titration.

Several limitations of the present study require an in-depth discussion. The patients were treated in two different hospitals; therefore, several selection and management biases may have been present. Yet, the same inclusion criteria and management protocols were adopted. The study sample was small and with few incident seizure cases, preventing any statistical analysis. Nonetheless, considering the modest number of patients treated with CAR T-cell therapy currently, our study has one of the largest cohorts of ICANS. The PP-group had a significantly longer disease history, a higher number of failed oncological therapies, and more frequently received Axi-cel as CAR T-cell product. Most patients who received primary prophylaxis were more frequently evaluated with EEG. Finally, a few patients from the hospital with pre-emptive ASM internal protocol were deliberately treated with primary prophylaxis by the treating physicians because they were considered at higher risk of developing neurotoxicity. Collectively, these latter limitations reflected a higher risk of developing seizures and a higher diagnostic sensitivity in the PP group. Currently, there is no evidence to support ASM primary prophylaxis in medicine except in patients with severe traumatic brain injury during the early post-traumatic period [28]. Therefore, ASMs are usually started only as a secondary prevention measure in patients with epilepsy [29]. The International League Against Epilepsy (ILAE) defines epilepsy as a disease with a tendency toward developing a seizure of at least 60% over the next ten years [30].

In this context, the risk of developing a seizure following CAR T-cell therapy is extremely high—up to 30% in the subsequent 7–10 days [9]. Therefore, advocating for short-term primary prophylaxis in CAR T-cell therapy in this population might be reasonable, especially in patients with identifiable risk factors for developing ICANS. Additionally, the initiation of anti-seizure medication before CAR T-cell infusion enables a gradual titration and dosage adjustment based on plasma levels, thereby mitigating potential side effects.

## Conclusions

Haematological patients undergoing anti-CD19 CAR T-cell therapy experience a temporary heightened susceptibility to seizures and status epilepticus. Administration of anti-seizure medications as either a primary or pre-emptive prophylactic measure is safe and effective in this population. Given the potential implications for patient care, a comprehensive randomized clinical trial would be necessary to determine the optimal anti-seizure medication strategy for this specific patient population. Yet, the optimal safety and efficacy profile of the short-term primary prophylaxis regimen might be sufficient to advocate for its widespread use, especially in the absence of reliable seizure-risk stratification scores. It is noteworthy that young females affected by PMBCL might present a higher predisposition to develop epileptic events.

### Supplementary Information

Below is the link to the electronic supplementary material.

Supplementary file1 (DOCX 22 KB)

## Data Availability

Data associated with this scientific article have been deposited in the open repository Zenodo, and they will be available upon appropriate request.
