# Correction to: Primary vs. pre-emptive anti-seizure medication prophylaxis in anti-CD19 CAR T-cell therapy

**DOI:** 10.1007/s10072-024-07510-y

**Published:** 2024-04-04

**Authors:** Umberto Pensato, Federica Pondrelli, Chiara de Philippis, Gian Maria Asioli, Alessandra Crespi, Alessandro Buizza, Daniele Mannina, Beatrice Casadei, Enrico Maffini, Laura Straffi, Simona Marcheselli, Pier Luigi Zinzani, Francesca Bonifazi, Maria Guarino, Stefania Bramanti

**Affiliations:** 1 Department of Biomedical Sciences, Humanitas University, Via Rita Levi Montalcini 4, 20072 Pieve Emanuele, Milan Italy; 2 IRCCS Humanitas Research Hospital, Via Manzoni 56, 20089 Rozzano, Milan Italy; 3 Department of Biomedical and Neuromotor Sciences (DIBINEM), University of Bologna, Bologna, Italy; 4 IRCCS Istituto Delle Scienze Neurologiche Di Bologna, Bologna, Italy; 5 BMT and Cell Therapy Unit, Humanitas Cancer Center, IRCCS Humanitas Research Hospital, Rozzano, Milan Italy; 6 IRCCS Azienda Ospedaliero-Universitaria Di Bologna, Istituto Di Ematologia “Seràgnoli”, Bologna, Italy; 7 Dipartimento Di Scienze Mediche E Chirurgiche, Università Di Bologna, Bologna, Italy

**Correction to: Neurological Sciences (2024)**

10.1007/s10072-024-07481-0

The original article contains an error in author affiliations. The corrections needed for the article are the following:

1. Alessandra Crespi and Alessandro Buizza require double affiliations (Affiliations 1 and 2, instead of Affiliation 1 only).
2. Stefania Bramanti requires a single affiliation (only Affiliation 5, and not Affiliation 2 and 5).

Affiliations are now updated here.

The original article has been corrected.

