# Supplementary material for: Primary vs. pre-emptive anti-seizure medication prophylaxis in anti-CD19 CAR T-cell therapy
Source: Neurol Sci. 2024 Mar 21;45(8):4007–14. doi: 10.1007/s10072-024-07481-0 (PMC11255041; doi:10.1007/s10072-024-07481-0)
Supplement: Supplementary file 1 — Supplementary file1 (DOCX 22 KB) [file 10072_2024_7481_MOESM1_ESM.docx]

**Supplementary Materials**

Patient 1 (PET-group)
A 31-year-old female affected by refractory PMBCL was treated with anti-CD19 CAR T-cell (Axi-cel) following pembrolizumab as a bridge therapy. Two days later, she developed a persistent CRS (grade 1) responsive to tocilizumab. On the fifth day, she developed bilateral postural myoclonus and global aphasia. Brain MRI with contrast was unremarkable, whereas continuous EEG showed a frontally predominant rhythmic delta activity with superimposed sharp waves and evolving morphological changes consistent with non-convulsive status epilepticus (ICANS grade 4). She was treated with multiple anti-seizure medications (levetiracetam, lacosamide, valproic acid), intravenous anaesthetics (propofol, ketamine, and midazolam), and high-dose intravenous steroids with no resolution. Therefore, she was diagnosed with super-refractory status epilepticus and treated with high-dose anakinra (100mg q6h). After a few days, a dramatic electro-clinical response was observed with no residual neurological deficit. After a few months, all the ASMs were discontinued. She had no neurological sequelae and complete oncological remission after six months of follow-up.

Patient 2 (PET-group)
A 52-year-old man affected by refractory DCBCL was treated with anti-CD19 CAR T-cell therapy (Tisa-cel). After 24 hours, he developed a persistent CRS (grade 1) responsive to tocilizumab. After ten days after the infusion, the patient was discharged home. On the fourteenth day, the patient presented a tonic-clonic seizure. Therefore, the patient was readmitted to the hospital and treated with five days of dexamethasone and anti-seizure medication (LEV 750mg bid). Brain MRI and EEG were unremarkable. He never presented other seizures or any neurological manifestation. After two months, the ASM was tapered and discontinued. He had no neurological sequelae and complete oncological remission after a one-year follow-up.

Patient 3 (PET-group)

A 31-year-old female affected by refractory PMBCL was treated with anti-CD19 CAR T-cell therapy (Axi-cel) after bridging with pembrolizumab. After 24 hours, she developed persistent CRS (grade 1) responsive to tocilizumab. On the seventh day, she experienced a tonic-clonic seizure followed by a persistent decrease in her level of consciousness (ICANS grade 4). She was admitted to the ICU and treated with levetiracetam (3g/day) and high-dose intravenous steroids. Two days later, there was a complete electro-clinical resolution. Brain MRI with contrast was unremarkable and a few days after the patient was discharged home.

She had complete oncological remission and no neurological sequelae.

Patient 4 (PRO-group)

A 33-year-old woman affected by refractory PMBCL was treated with anti-CD19 CAR-T therapy (Axi-cel) after bridging with pembrolizumab. The following day, she developed a grade II CRS refractory to tocilizumab. On the fourth day, she developed an acute encephalopathy with aphasia, ocular flutter, and limb myoclonus. Due to a decrease in consciousness, she was admitted to the ICU, high-dose methylprednisolone (1000 mg/q24h) was started and levetiracetam increased (3g/day). About 20 hours later, she had a tonic-clonic seizure and sedation with propofol was started. At anesthetic withdrawal (day +5), the patient was stuporous and with complete aphasia (ICANS 4). Brain MRI with contrast was normal, and EEG showed a severe slowing of background activity. Anakinra (100 mg/q12h) and Siltuximab (11 mg/kg, single dose) were started with complete neurological remission in the next four days. The steroid was progressively reduced and Anakinra was discontinued after seven days. She had a complete oncological response after one year and no neurological sequelae.

Patient 5 (PRO-group)

A 63-year-old man affected by refractory DLBCL was treated with anti-CD19 CAR T-cell (Axi-cel). A few hours later, he developed a grade I CRS responsive to Tocilizumab. On the fourth day, he developed acute-onset aphasia, tremor, and myoclonus (ICANS grade 3). Intravenous dexamethasone (20 mg/q6h) was started, and the patient was transferred to the ICU. About five hours later, he presented three brief tonic-clonic seizures; thus levetiracetam was increased (3g/day) and immunotherapy escalated to intravenous high-dose steroid and Siltuximab (11 mg/kg, single dose). EEG disclosed a mild slowing of background activity without epileptiform discharges; brain MRI showed bi-thalamic T2-FLAIR hyperintensity. No more seizure was reported and neurological status progressively improved in the next five days, steroid was gradually withdrawn. At one year follow-up, he didn’t experience any neurological sequelae or seizure; the hematological disease showed only a partial response.
